# A validation of presepsin levels in kidney dysfunction patients: four case reports

**DOI:** 10.1186/s40560-014-0063-2

**Published:** 2014-12-03

**Authors:** Atsushi Kotera, Katsuyuki Sagishima, Takahiro Tashiro, Daisuke Niimori, Hidenobu Kamohara, Yoshihiro Kinoshita

**Affiliations:** Department of Intensive Care Medicine, Kumamoto University Hospital, 1-1-1 Honjo, Chuo-ku, Kumamoto City, Kumamoto Prefecture 860-8556 Japan

**Keywords:** Acute kidney injury, Chronic kidney disease, Presepsin, Sepsis

## Abstract

Here, we measured presepsins (PSPs) in four patients with acute kidney injury (AKI) or chronic kidney disease (CKD) and discuss the relationship between PSP and kidney dysfunction.

Case 1: an 83-year-old man was admitted to the ICU to manage postoperative respiratory failure with AKI. He had undergone resection for rectal cancer and ileal conduit replacement. On day 1 in the ICU, *Escherichia coli* (*E. coli*) was isolated by urine culture. PSP level (pg/ml) on day 2 was 2,745 without elevation of other conventional biomarkers. On day 6, the patient was diagnosed with severe sepsis, and *E. coli* was isolated by blood culture. By then, PSP had risen to 3,977, along with elevation of other conventional biomarkers. His kidney function recovered gradually after continuous administration of hemodiafiltration; however, PSP continued to rise up to 6,051, along with high systemic inflammatory response syndrome (SIRS) and Acute Physiology and Chronic Health Evaluation (APACHE) II values. The patient expired on day 13 due to multiple organ failure. Case 2: a 78-year-old woman with CKD on hemodialysis (HD) was admitted to the ICU after cardiovascular surgery. Continuous HD was administered postoperatively, and PSP ranged from 1,473–1,870 without signs of sepsis. Temporary elevation of other conventional biomarkers was observed postoperatively. Case 3: a 74-year-old woman with CKD on HD was admitted to the ICU after neurosurgery. She underwent intermittent HD postoperatively, and PSP ranged from 1,240–1,935 without sepsis symptoms. Temporary elevation of other conventional biomarkers was observed postoperatively. Case 4: a 62-year-old man with CKD was admitted to the ICU to control gastrointestinal bleeding. PSP was 606 without signs of infection or elevation of other conventional biomarkers. In cases 2, 3, and 4, bacteria were not isolated in blood cultures. Patients’ clinical prognoses were good, with low or moderate SIRS and APACHE II scores.

PSP in kidney dysfunction patients will be high despite non-infectious conditions. Therefore, evaluation of PSP in kidney dysfunction patients will be difficult. Further investigation is needed to clarify the relationship between PSP and kidney dysfunction.

## Correspondence/findings

### Background

Presepsin, a 13-kDa protein, constitutes a truncated N-terminal fragment of CD 14, the receptor for lipopolysaccharide-lipopolysaccharide binding protein complexes [1]. It has been shown to be a more sensitive and specific marker for the diagnosis of sepsis than other conventional biomarkers [2]. When the cutoff value of presepsin for discrimination of sepsis and non-sepsis is 600 pg/ml, the clinical sensitivity is 87.8% and the clinical specificity is 81.4% [3].

Median presepsin (PSP (pg/ml)) level was reported to be 130 in healthy volunteers, 212 in systemic inflammatory response syndrome (SIRS) patients, 325 in sepsis patients, 787 in severe sepsis patients, and 1,084 in septic shock patients [4]. On the other hand, it has been reported that PSP tends to increase in kidney dysfunction patients [5]. In the present study, we measured PSPs in four patients with acute kidney injury (AKI) or chronic kidney disease (CKD) and discuss the relationship between PSP and kidney dysfunction.

### Case presentation

#### Materials and methods

We measured PSP with a PATHFAST analyzer (Mitsubishi Chemical Medience Corporation, Tokyo, Japan). Procalcitonin (PCT) and C-reactive protein (CRP) levels were measured using the same blood sample, and we also calculated SIRS and Acute Physiology and Chronic Health Evaluation (APACHE) II scores. For this report, kidney dysfunction was defined as an estimated glomerular filtration rate (e GFR) of less than 60 ml/min/m^2^ [6]. Written informed consent was obtained from all of the four patients for publication of case reports and any accompanying images. This retrospective study was approved by the Ethical Committee of Kumamoto University Hospital.

#### Cases

Case 1: an 83-year-old man was admitted to the ICU in order to manage postoperative respiratory failure with AKI. He had previously undergone resection of rectal cancer and ileal conduit replacement. Clinical data from his ICU stay are shown in Table 1 (case 1). On his 1st day in the ICU, *Escherichia coli* (*E. coli*) was isolated by urine culture and normal florae were isolated by oral culture (Table 2 (case 1)). PSP measured on the 2nd day was 2,745 without PCT or CRP elevation. However, on the 6th day, severe sepsis set in and *E. coli* was isolated by blood culture. PSP was elevated to 3,977, and PCT and CRP had also increased. The patient’s kidney function recovered gradually with continuous hemodiafiltration; however, PSP rose to 6,051 on the 9th day, along with high SIRS and APACHE II values. The patient did not survive on the 13th day due to multiple organ failure.

**Table 1:** **Follow-up of PSP, CRP, procalcitonin, e GFR, creatinine, SIRS score, and APACHE II score during ICU stay**

Case 1	Day 2	Day 6	Day 7	Day 9	Day 10
PSP (pg/ml)	2,7453,9773,9406,0515,163				
CRP (mg/dl)	0.35	23.44	23.07	17.91	19.37
Procalcitonin (ng/ml)	0.51	1.11	------	------	------
e GFR (ml/min/m^2^)	28	30	44	71	70
SIRS score	2	3	2	3	3
Case 2	Day 1	Day 2	Day 3	Day 4	
PSP (pg/ml)	1,7511,8701,4731,660				
CRP (mg/dl)	7.45	14.99	14.28	6.42	
Procalcitonin (ng/ml)	-----	------	1.33	-----	
e GFR (ml/min/m^2^)	8	6	6	14	
SIRS score	2	2	2	1	
Case 3	Day 1	Day 3	Day 5	Day 7	
PSP (pg/ml)	1,2441,2401,5121,935				
CRP (mg/dl)	1.54	23.09	13.68	5.75	
Procalcitonin (ng/ml)	------	0.76	------	-------	
e GFR (ml/min/m^2^)	3	3	5	7	
SIRS score	2	2	2	2	
Case 4	Day 1				
PSP (pg/ml)	606				
CRP (mg/dl)	0.77				
Procalcitonin (ng/ml)	1.06				
e GFR (ml/min/m^2^)	33				
SIRS score	1				
APACHE II score	13				

*PSP* presepsin level, *CRP* C-reactive protein, *e GFR* estimated glomerular filtration rate, *SIRS* systemic inflammatory response syndrome, *APACHE II* Acute Physiology and Chronic Health Evaluation II.

**Table 2:** **Culture tests performed on the 1st day in the ICU**

	**Oral cavity**	**Urine**	**Sputum**	**Blood**
Case 1				
Isolated bacteria	*Corynebacterium striatum* (10^6^)	*Escherichia coli* (10^6^)	None	None^a^
Case 2				
Isolated bacteria	*Corynebacterium striatum* (10^6^)	None	None	None
Case 3				
Isolated bacteria	*Neisseria* species (10^7^)	None	None	None
	*Streptococcus* species (10^6^)			
Case 4				
Isolated bacteria	*Streptococcus* species (10^6^)	None	None	None
	*Neisseria* species (10^6^)			
	*Micrococcus* species (10^6^)			

^a^ *E. coli* was isolated (4 days later) by both venous blood sample and arterial blood sample.

Case 2: a 78-year-old woman with CKD on hemodialysis (HD) was admitted to the ICU after a coronary artery bypass graft under cardiopulmonary pump. Continuous HD was undergone postoperatively, and PSP ranged from 1,473–1,870 without sepsis symptoms. Clinical data from her ICU stay are presented in Table 1 (case 2). Temporary elevation of CRP or PCT was observed; however, SIRS and APACHE II values gradually decreased postoperatively. Bacteria were not isolated in blood culture, and normal florae were isolated in oral culture (Table 2 (case 2)). Postoperative complications were not observed, and the patient’s clinical prognosis was good.

Case 3: a 74-year-old woman with CKD on HD was admitted to the ICU after surgical clipping for intracranial arterial aneurysm. Intermittent HD was undergone postoperatively, and PSP ranged from 1,240–1,935 without sepsis symptoms. Clinical data from her ICU stay are listed in Table 1 (case 3). Temporary elevation of CRP or PCT was also observed, while SIRS and APACHE II values did not change postoperatively (Table 1 (case 3)). Bacteria were not isolated in blood culture, and normal florae were isolated in oral culture (Table 2 (case 3)). Postoperative complications were not observed, and the patient’s clinical prognosis was good.

Case 4: a 62-year-old man with CKD was admitted to the ICU in order to control gastrointestinal bleeding. Initial PSP was 606 without infection. Clinical data on his 1st day in the ICU are presented in Table 1 (case 4). His CRP was not elevated and PCT was elevated only slightly, while low SIRS and APACHE II values were observed. Bacteria were not isolated in blood culture, and normal florae were isolated in oral culture (Table 2 (case 4)). No signs of infection were observed, and the patient’s clinical prognosis was good.

## Discussion

Presepsin is a 13-kDa protein; hence, it is usually filtered by the glomerulus and reabsorbed and completely catabolized within proximal tubular cells. Therefore, in kidney dysfunction patients, PSP tends to elevate due to the low clearance of glomerular and renal tubular cells [5]. Endo et al*.* [3] demonstrated that PSPs in two CKD patients without sepsis were 9,036 and 1,362, while their PCTs were 0.525 and 0.382 ng/ml, respectively. Zou et al. [7] reported that the median PSP in non-sepsis kidney dysfunction patients was 1,607 (range, 454–8,516) and those in sepsis kidney dysfunction patients was 1,523 (range, 293–16,764); they also mentioned that there was no significant difference between the two groups. Our four case studies present four patients who revealed high PSP values without elevation of other conventional biomarkers or symptoms of sepsis. In cases 1, 2, and 3, PSPs measured before initiation of renal replacement therapy (RRT) were almost the same as those measured during RRT (data not shown). We used the same type of polysulfone membrane (Asahi Kasei Medical Co., Ltd., Tokyo, Japan) for RRT in all cases, and it was considered that the effect of RRT with polysulfone membrane might be negligible for PSP.

The AKI patient (case 1) demonstrated an extremely high PSP value compared to the CKD patients (cases 2, 3, and 4). Behnes et al. [8] reported that PSP correlated with creatinine levels in both AKI patients and CKD patients. In general, PSP did not correlate with the primary sites of bacteria, such as urinary tract, blood, lung, or oral [9]. In case 1, in addition to oral bacterial florae, *E. coli* was initially isolated in the patient’s urine culture. Severe sepsis due to *E. coli* was detected 4 days later. Therefore, PSP’s own early response to bacterial infection might have contributed to the extremely high PSP value. In addition, low clearance of PSP due to kidney dysfunction might also contribute to extremely high values. Indeed, Endo et al. [3] reported that, in 12-PSP-false-positive patients, five were diagnosed with infections in the 7 days after admission. Therefore, we could not deny the possibility that the initial PSP value in case 1 was correlated with the infectious condition induced by *E. coli*. On the other hand, in cases 2, 3, and 4, normal bacterial florae were isolated by oral culture. It was considered that, in CKD patients, even normal florae in the oral cavity might generate high values of PSP due to the low clearance of glomerular and renal tubular cells.

The differentiation between sepsis and non-sepsis using PSP measurement may be difficult in kidney dysfunction patients. During the follow-up period, the AKI patient with sepsis (case 1) revealed an extremely high rate of increase of PSP value compared to the CKD patients without sepsis (cases 2 and 3). Hence, in kidney dysfunction patients, it is considered that temporary PSP measurement may not be useful for the diagnosis of sepsis and continuous PSP measurement will be recommended for discrimination between sepsis and non-sepsis.

## Conclusions

PSP in kidney dysfunction patients will be high despite non-infectious conditions. Therefore, care should be taken when infection is diagnosed using PSP in kidney dysfunction patients. Further investigation will be needed to clarify the relationship between PSP and kidney dysfunction patients without infection.

### Consent

Written informed consent was obtained from patients for publication of these case reports and any accompanying images.
